# Variation in electroencephalography and neuroimaging for children receiving extracorporeal membrane oxygenation

**DOI:** 10.1186/s13054-022-04293-6

**Published:** 2023-01-17

**Authors:** Joseph G. Kohne, Graeme MacLaren, Renée A. Shellhaas, Giulia Benedetti, Ryan P. Barbaro

**Affiliations:** 1 Division of Pediatric Critical Care Medicine, Department of Pediatrics, University of Michigan, Ann Arbor, USA; 2 Susan B. Meister Child Health Evaluation and Research Center, University of Michigan School of Medicine, Ann Arbor, USA; 3 Cardiothoracic Intensive Care Unit, National University Health System, Singapore, Singapore; 4 Division of Pediatric Neurology, Department of Pediatrics, University of Michigan, Ann Arbor, USA; 5 Department of Neurology, Seattle Children’s Hospital and University of Washington, Seattle, USA

**Keywords:** Extracorporeal membrane oxygenation, Neuroimaging, Seizures, Stroke, Brain injuries, Hospitals, Pediatric, Electroencephalography

## Abstract

**Background:**

Seizures, strokes, and intracranial hemorrhage are common and feared complications in children receiving extracorporeal membrane oxygenation (ECMO) support. Researchers and clinicians have proposed and deployed methods for monitoring and detecting neurologic injury, but best practices are unknown. We sought to characterize clinicians’ approach to electroencephalography (EEG) and brain imaging modalities in children supported by ECMO.

**Methods:**

We performed a retrospective observational cohort study among US Children’s Hospitals participating in the Pediatric Health Information System (PHIS) from 2016 to 2021. We identified hospitalizations containing ECMO support. We stratified these admissions by pediatric, neonatal, cardiac surgery, and non-cardiac surgery. We characterized the frequency of EEG, cranial ultrasound, brain computed tomography (CT), magnetic resonance imaging (MRI), and transcranial Doppler during ECMO hospitalizations. We reported key diagnoses (stroke and seizures) and the prescription of antiseizure medication. To assess hospital variation, we created multilevel logistic regression models.

**Results:**

We identified 8746 ECMO hospitalizations. Nearly all children under 1 year of age (5389/5582) received a cranial ultrasound. Sixty-two percent of the cohort received an EEG, and use increased from 2016 to 2021 (52–72% of hospitalizations). There was marked variation between hospitals in rates of EEG use. Rates of antiseizure medication use (37% of hospitalizations) and seizure diagnoses (20% of hospitalizations) were similar across hospitals, including high and low EEG utilization hospitals. Overall, 37% of the cohort received a CT and 36% received an MRI (46% of neonatal patients). Stroke diagnoses (16% of hospitalizations) were similar between high- and low-MRI utilization hospitals (15% vs 17%, respectively). Transcranial Doppler (TCD) was performed in just 8% of hospitalizations, and 77% of the patients who received a TCD were cared for at one of five centers.

**Conclusions:**

In this cohort of children at high risk of neurologic injury, there was significant variation in the approach to EEG and neuroimaging in children on ECMO. Despite the variation in monitoring and imaging, diagnoses of seizures and strokes were similar across hospitals. Future work needs to identify a management strategy that appropriately screens and monitors this high-risk population without overuse of resource-intensive modalities.

**Supplementary Information:**

The online version contains supplementary material available at 10.1186/s13054-022-04293-6.

## Introduction

Extracorporeal membrane oxygenation (ECMO) is used to support children with severe heart or lung failure. Neurologic injury sustained during ECMO support is one of the most feared complications for clinicians and families [1]. Patients on ECMO support are at risk of brain injury secondary to hypoperfusion, thromboembolism, hemorrhage, and reperfusion. A recent prospective multicenter observational study reported an incidence of 4% and 16% for ischemic and hemorrhagic neurologic complications of children on ECMO, respectively [2]. Also, single-center studies have demonstrated seizures in 17–40% of ECMO patients, many of which were only detectable on electroencephalography (EEG) [3–6].

Clinicians deploy several strategies to identify brain injury in children because of the high risk of neurologic injury. For infants with an open fontanelle, cranial ultrasonography has been widely used and has the benefit of being portable and without radiation exposure [7–9]. Imaging such as computed tomography (CT) and magnetic resonance imaging (MRI) may be used when there is suspicion of brain injury, but CT includes radiation exposure, and MRI is not possible during ECMO support. EEG can be used to detect seizures, monitor sedation, and may detect new focal abnormalities in the presence of ischemic or hemorrhagic brain injury [10, 11]. Less common neuromonitoring techniques such as transcranial Doppler (TCD) have also been suggested to monitor neurologic injury in patients receiving ECMO support [12–14].

We sought to characterize the trends and hospital variation in the utilization of EEG and neuroimaging during ECMO hospitalizations among US children's hospitals using the Pediatric Health Information System (PHIS). This database allows for the assessment of current practices across US tertiary children's hospitals.

## Materials and methods

This study was determined to be not regulated as human subjects research by the University of Michigan Institutional Review Board and all ethical standards were followed (HUM00212344, 1/28/22). Data for this study were obtained from the Pediatric Health Information System (PHIS), an administrative database that contains inpatient, emergency department, ambulatory surgery, and observation encounter-level data from not-for-profit, tertiary care pediatric hospitals in the USA [15]. These hospitals are affiliated with the Children’s Hospital Association (Lenexa, KS). Data quality and reliability are assured through a joint effort between the Children's Hospital Association and participating hospitals. For the purposes of external benchmarking, participating hospitals provide discharge/encounter data including demographics, diagnoses, and procedures. Nearly all of these hospitals also submit resource utilization data (e.g., pharmaceuticals, imaging, and laboratory) into PHIS. Data are de-identified at the time of data submission, and data are subjected to reliability and validity checks before being included in the database. For this study, we included data from the 47 hospitals that contributed full data to PHIS at the time of the data request.

We included ECMO hospitalizations with a discharge date from January 1st, 2016 through December 31st, 2021. Hospitalizations were identified through ICD-10 procedure codes for ECMO (Additional file 1: Table S1). To allow subgroup analyses, we identified neonatal hospitalizations and hospitalization that included cardiac surgery. We defined neonatal admissions as those with a patient age of 28 days or less at the time of the first ECMO charge. We identified hospitalizations that included cardiac surgery through Risk adjustment for congenital heart surgery-2 (RACHS-2) model for ICD-10(c). RACHS-2 is an empirically derived, risk adjustment model that has been validated in two separate administrative data sources and compared to locally held clinical registry data [16].

We identified the following modalities through clinical transaction classification (CTC) codes used by PHIS: EEG, cranial ultrasound, head CT, brain MRI, and transcranial Doppler. We secondarily assessed the rates of seizure and stroke diagnoses and prescription of antiseizure medication. We determined stroke and seizure diagnoses through the presence of diagnosis codes [17–20]. We report whether a patient was prescribed an antiseizure medication using CTC codes, excluding medications most likely to be used for sedation including benzodiazepines (Additional file 1: Table S1). Complex chronic conditions were determined according to complex chronic conditions classification software v2 developed by Feudtner et al. [21].

For each patient who received ECMO, the presence and number of charges for EEG and each imaging modality was recorded. We reported the proportion of patients who received each modality during the ECMO hospitalization. We also noted the median and interquartile range (IQR) for the frequency of each modality by patient who received the study. We reported patient outcomes of ECMO duration, hospital length of stay, and hospital mortality. We determined ECMO duration through the days of consecutive ECMO charges. If there was one day between ECMO charges, it was counted as a consecutive ECMO run. Bivariate comparisons were conducted through Pearson’s Chi-squared for categorical variables and Wilcoxon rank-sum for continuous variables. We tested for trends across years using a nonparametric test for trend [22].

For MRI and EEG, we grouped hospitals into quintiles based on the utilization of each modality. We report the frequency of a stroke diagnosis for each MRI quintile. We report the frequency of seizure diagnosis and antiseizure medication use for each EEG quintile. To assess variability between hospitals, we created a multilevel logistic regression model where hospitalizations were nested within hospitals, with the outcome of whether or not a patient received the modality. We modeled PHIS hospital as a random effect. For TCD, EEG, and head CT, we adjusted for patient-level covariates of patient age, duration of ECMO support, discharge year, and receipt of cardiac surgery a priori. For MRI, we also adjusted for patient in-hospital mortality, to account for patients needing to survive to decannulation from ECMO support to receive an MRI. To describe center-level variation, we reported the intraclass correlation coefficient and plotted the predicted probability of receiving each modality by center in a caterpillar plot [23, 24]. Analyses were completed in Stata (16.1, StataCorp LLC, College Station, TX).

## Results

### Cohort description

We identified 8746 hospitalizations among 8633 patients who received ECMO at 47 children's hospitals in the PHIS database. Forty-six percent (4007/8746) of the cohort were neonatal admissions (age < 28 days).Cohort characteristics are shown in Table 1. The median age of the non-neonatal admissions was 2 years (IQR 0–11 years). Cardiac surgery occurred during 36% (3163/8746) of hospitalizations, 39% of which were neonates (1244/3163). The frequencies of EEG and neuroimaging studies among neonates/children and cardiac surgery/non-cardiac surgery hospitalizations are shown in Table 2. In-hospital mortality occurred in 39% of admissions. The rates of EEG and neuroimaging modalities in those infants and children who survived to discharge and comparing those who did and did not survive to discharge are shown in Additional file 1: Tables S2 and S3. The median hospital length of stay was 38 days (IQR 18–77 days). Among 6717 hospitalizations with ECMO charges across multiple days, the median duration of ECMO support was 5 days (IQR 3–10 days).

**Table 1:** Cohort characteristics of ECMO Hospitalization among children and neonates

Characteristic	Total	Children	Neonates	*p* value
	*N* = 8746	*N* = 5188	*N* = 3,558	
Age (years), median (IQR)	0 (0–4)	2 (0–11)	–	–
Female, *n* (%)	3986 (46%)	2458 (47%)	1528 (43%)	< 0.001
Complex chronic condition^a^, *n* (%)				
Cardiovascular	6317 (72%)	3932 (76%)	2385 (67%)	< 0.001
Gastrointestinal	2455 (28%)	1568 (30%)	887 (25%)	< 0.001
Hematologic or immunologic	1089 (12%)	857 (17%)	232 (7%)	< 0.001
Malignancy	381 (4%)	350 (7%)	31 (1%)	< 0.001
Metabolic	1911 (22%)	1581 (30%)	330 (9%)	< 0.001
Neurologic and neuromuscular	1981 (23%)	1481 (29%)	500 (14%)	< 0.001
Congenital or genetic defect	1899 (22%)	765 (15%)	1,134 (32%)	< 0.001
Renal and urologic	2005 (23%)	1186 (23%)	819 (23%)	0.86
Respiratory	2010 (23%)	1048 (20%)	962 (27%)	< 0.001
Premature And neonatal	3263 (37%)	613 (12%)	2650 (74%)	< 0.001
Technology dependent	3950 (45%)	2696 (52%)	1254 (35%)	< 0.001
Transplant	582 (7%)	551 (11%)	31 (1%)	< 0.001
Cardiac Surgery^b^, *n* (%)	3163 (36%)	1919 (37%)	1244 (35%)	0.053
RACHS-1	176 (2%)	132 (3%)	44 (1%)	< 0.001
RACHS-2	572 (7%)	422 (8%)	150 (4%)	< 0.001
RACHS-3	852 (10%)	700 (13%)	152 (4%)	< 0.001
RACHS-4	833 (10%)	446 (9%)	387 (11%)	< 0.001
RACHS-5	730 (8%)	219 (4%)	511 (14%)	< 0.001
Length Of stay (days), median (IQR)	38 (18–77)	36.5 (16–75)	39 (21–77)	< 0.001
Billed charges (US dollars), median (IQR)	1,161,340 (609474.3–2,197,633)	1,185,444 (579129.1–2,367,075)	1,136,905 (637995.1–1,982,730)	0.045
Hospital Mortality, *n* (%)	3401 (39%)	2079 (40%)	1322 (37%)	0.006

^a^Complex chronic conditions classification v2 [21]

^b^Risk Stratification for Congenital Heart Surgery for ICD-10 Administrative Data (RACHS-2)[16]

**Table 2:** Frequency of EEG/Neuroimaging modalities among age and etiology subgroups

Modality	Total	Age group			Etiology		
		Children	Neonates	*p*	Non-cardiac surgery	Cardiac Surgery	*p*
	*n* = 8746	*n* = 5188	*n* = 3558		*n* = 5583	*n* = 3163	
Magnetic resonance imaging	3130 (36%)	1479 (29%)	1651 (46%)	< 0.001	2164 (39%)	966 (31%)	< 0.001
Transcranial Doppler	667 (8%)	313 (6%)	354 (10%)	< 0.001	394 (7%)	273 (9%)	0.008
Computed tomography	3267 (37%)	2568 (49%)	699 (20%)	< 0.001	1868 (33%)	1399 (44%)	< 0.001
Cranial ultrasound	5510 (63%)	2001 (39%)	3509 (99%)	< 0.001	3126 (56%)	2384 (75%)	< 0.001
Under age 1 (*n* = 5582)	5389 (97%)	–	–	–	3048 (96%)	2341 (97%)	0.005
Electroencephalography	5450 (62%)	3389 (65%)	2061 (58%)	< 0.001	3299 (59%)	2151 (68%)	< 0.001

### Electroencephalography

**Fig. 1:**
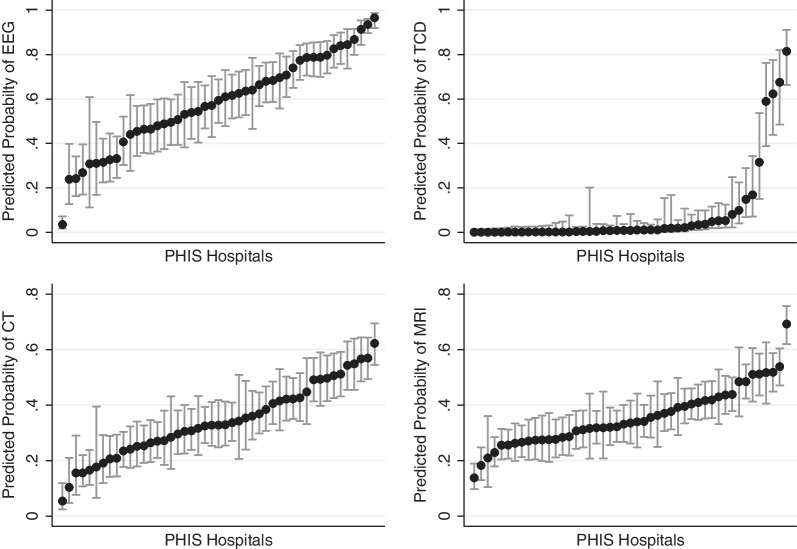
Variation in use of electroencephalography (EEG) and neuroimaging across Pediatric Health Information System (PHIS) hospitals. TCD: transcranial Doppler; CT: computed tomography; MRI: magnetic resonance imaging. The dots are the predicted estimate for the use of each modality by hospital, adjusted for patient age, year of discharge, duration of ECMO support, and receipt of cardiac surgery. Hospital survival was also included in the model for MRI. Error bars represent the 95% confidence interval

Sixty-two percent (5450/8746) of the cohort underwent EEG during the hospitalization (58% of neonates, 65% of children > 28 days) (Additional file 1: Table S4). Of patients who received an EEG, there was a median three charges/patient (IQR 2–6). The first EEG was performed a median 1 day following the first ECMO charge (IQR 0–2 days). Hospitalizations including cardiac surgery were more likely to have an EEG (68% vs. 59%, *p* < 0.001). There was an increase in EEG utilization from 52% in 2016 to 72% in 2021 (*p* < 0.001). After adjustment for patient age, year of discharge, duration of ECMO support, and receipt of cardiac surgery, the hospital accounted for 30% of the residual variation (Fig. 1, Additional file 1: Table S5).

**Fig. 2:**
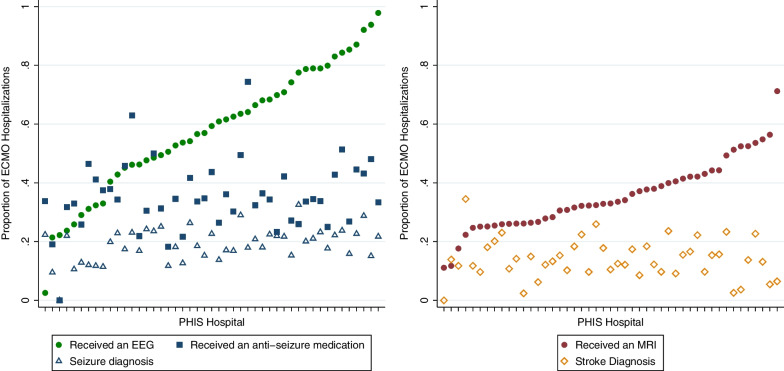
In the first frame, we present the unadjusted rates of EEG, antiseizure medication, and seizure diagnosis among ECMO hospitalizations. The proportion of ECMO hospitalizations at each PHIS hospital which included an EEG, antiseizure medication, or seizure diagnosis is represented on the *y*-axis. Each individual hospital is presented on the *x*-axis, sorted from lowest EEG utilization to the highest. In the second frame, we similarly present the rates of MRI and stroke diagnosis among ECMO hospitalizations

In the entire cohort, 37% (3195/8746) of hospitalizations included prescription of an antiseizure medication (Fig. 2). Of the 5450 hospitalizations that included an EEG, 46% included a prescription for an antiseizure medication (of note, 20% [672/3296] of hospitalizations that had no EEG did include an antiseizure medication, *p* < 0.001). Twenty percent (1730/8746) of hospitalizations included a seizure diagnosis code (28% of those who received an EEG). Between the hospitals in the highest and lowest EEG utilization quintiles, rates of antiseizure medications were 40% and 36% (*p* = 0.035) and rates of seizure diagnosis were 20% and 16% respectively (*p* = 0.001) (Table 3). The medication types and frequency of those who did and did not receive an EEG during the hospitalization are shown in Additional file 1: Table S6.

**Table 3:** Proportion of stroke diagnosis by MRI quintile, and seizure diagnosis, stroke diagnosis, and antiseizure medication use by EEG quintile. PHIS hospitals were divided into quintiles based on the rates of MRI and EEG use during ECMO hospitalizations. The rates of stroke diagnoses, seizure diagnoses, and antiseizure medication use for the hospitals within the quintiles are reported below

MRI/EEG utilization quintile						
Measure	Total	1st (lowest utilization)	2nd	3rd	4th	5th (highest utilization)
*MRI*						
Stroke diagnosis	16% (1373/8746)	17% (211/1345)	13% (185/1409)	17% (290/1712)	15% (322/2080)	15% (240/1591)
*EEG*						
Antiseizure medication use	37% (3195/8746)	36% (489/1345)	37% (489/1310)	37% (642/1724)	33% (800/2429)	40% (775/1938)
Seizure diagnosis	20% (3195/8746)	16% (211/1345)	21% (269/1310)	20% (337/1724)	21% (517/2429)	20% (396/1938)
Stroke diagnosis	16% (1373/8746)	11% (151/1345)	13% (166/1310)	14% (246/1724)	19% (468/2429)	18% (342/1938)

### Cranial ultrasound and transcranial Doppler

Among children under 1 year of age, 97% (5376/5562) of hospitalizations included a cranial ultrasound (99% of neonates). For those who received a cranial ultrasound, they received a median of six ultrasounds [IQR 4–10].

TCD was completed in 8% (667/8746) of hospitalizations in the cohort, including 10% of neonates and 6% of children > 28 days. There was no trend in utilization of TCD across years (*p* = 0.07). Seventy-seven percent of the patients who received TCD were cared for at one of five centers. Nineteen percent (128/667) of those who received TCD were over 1 year of age.

### Computed tomography/magnetic resonance imaging

A head CT was performed in 37% (3267/8746) of hospitalizations (20% of neonatal hospitalizations and 50% of hospitalizations for children > 28 days). Utilization increased over time (34% in 2016 to 40% in 2021, *p* < 0.001). The hospital accounted for 14% of the residual variation after adjustment for patient age, year of discharge, duration of ECMO support, and receipt of cardiac surgery.

Brain MRI was obtained in 36% (3130/8746) of the cohort (46% of neonatal patients and 29% of children > 28 days, *p* < 0.001). Hospitalizations including cardiac surgery were less likely to include an MRI (31% vs 39%, *p* < 0.001). Children who received an EEG were more likely to receive an MRI than those that did not (41% vs 27%, *p* < 0.001). Of those who received an MRI, it was completed at a median 10 days following the last ECMO charge (IQR 3–27 days). When evaluating only those who survived to hospital discharge, 50% (2696/5345) of survivors (67% of neonates and 38% of children older than 28 days) received an MRI. There was no trend across years of MRI in survivors (*p* = 0.07). Between the hospitals in the highest and lowest MRI utilization quintiles, rates of stroke diagnosis were 15% and 17%, respectively (*p* = 0.09) (Table 3). After adjustment for patient age, year of discharge, duration of ECMO support, receipt of cardiac surgery, and a covariate of whether the patient died during the hospitalization, the hospital accounted for 9% of the residual variation.

## Discussion

In this analysis of children supported by ECMO at US Children's Hospitals, we found that there is significant variation in the use of EEG and neuroimaging modalities across hospitals, with the exception of cranial ultrasound, which was nearly universally applied in eligible infants. This center-level variation is especially pronounced in the use of EEG, which increased from 52% in 2016 to 72% in 2021. Among high and low EEG utilizers, the differences in rates of antiseizure medication use (36% vs 40%) and seizure diagnosis (16% vs 20%) were negligible. Similarly, there was a negligible difference in stroke diagnosis (17% vs 15%) between hospitals that were high MRI utilizers versus low utilizers.

The risk of neurologic injury during ECMO support is well known; however, the modalities and practices different centers use to monitor for and diagnose neurologic injury has not previously been reported. This study has several strengths. The PHIS database allows for an evaluation of practices across 47 children’s hospitals, has checks for reliability and validity, and has previously been used in pediatric critical care and ECMO research [25, 26]. The rates of stroke and seizure diagnoses seen in this cohort are consistent with the rates described in clinical research studies [2–4], supporting the accuracy of the diagnosis codes used.

There was significant variation among children’s hospitals in the application of EEG with increasing use observed during the study period. This increase in use may reflect increasing awareness of seizures in children supported by ECMO and practice change toward more proactive neuromonitoring [3, 6, 27, 28]. There did not seem to be a connection in our data between the rate of EEG use at a hospital and the diagnosis of seizures or use of antiseizure medication. EEG has utility beyond seizure diagnosis, and its possible centers with high EEG use are deploying EEG to monitor sedation, assess for evolving asymmetries or other patterns that suggest new brain injury, or monitor for other neurologic dysfunction [10]. We observed a median of three days of EEG charges; importantly, we were unable to determine if this was continuous EEG monitoring or intermittent monitoring. We additionally were unable to determine if any patients’ EEGs were processed to include amplitude-integrated EEG or other quantitative EEG measures.[29] Another important consideration in the application of EEG monitoring is the significant cost and resource burden associated with continuous monitoring [30, 31]. A better understanding of the risk factors and timeline for neurologic injury in patients supported by ECMO may enable more cost-effective monitoring strategies.

There were notable differences between the subgroups analyzed. Compared with older children, neonates were more likely to receive an MRI during the hospitalization (46% vs 29%) but less likely to receive a CT (20% vs 49%). ELSO guidelines recommend considering MRI in all neonates or children less than 2 years of age who receive ECMO support, because subtle neurologic deficits may not be as apparent in this cohort [32]. This difference in use may represent awareness or adherence to these guidelines, or general approach toward higher utility of MRI in smaller children or patients where the clinical exam is less reliable. Yet, the diagnosis of stroke was similar between the high- and low-MRI utilization hospitals, which does not support the idea that routine use of neuroimaging may uncover neurologic injury that is not clinically apparent during the acute hospital admission [33, 34].

Compared to non-cardiac surgery hospitalizations, cardiac surgery hospitalizations including ECMO support were more likely to receive a CT (44% vs 33%) but less likely to receive an MRI (31% vs 39%). In the Bleeding and Thrombosis on ECMO (BATE) study, a cardiac indication for support was associated with an increased risk of both hemorrhagic and thrombotic events [2] although notably subsequent analyses have not shown a relationship between specific cardiac diagnoses or procedures with bleeding risk [35]. These differences and the findings noted above highlight that the variation in EEG and neuroimaging on ECMO likely not only has inter-hospital variation but also significant intra-hospital variation as neonatologists, intensivists, cardiologists, and surgeons approach the monitoring and diagnosis of brain injury differently.

The use of bedside tests that can detect brain injury or conditions that place a patient at risk for brain injury is appealing. Prior studies suggest that near-infrared spectroscopy is being widely used for this purpose; however, its use was not captured in this dataset [36–38]. TCD has also been described for this purpose during ECMO support [13, 14, 39]. In this study, its use was limited to a small number of centers, where these noninvasive tests fit into the comprehensive neuromonitoring of a patient supported by ECMO should be a focus of future studies.

This study has important limitations. First, the use of billing data allowed for the characterization of practices across hospitals, but with outcomes limited to diagnosis codes, we are unable to accurately describe the results of the diagnostic tests. Similarly, we are unable to determine with accuracy whether the neurologic conditions described were acquired during the hospitalization or were pre-existing. Characterizing neurologic morbidities is essential in designing protocols for monitoring and screening. Second, the PHIS dataset contains admissions from US children’s hospitals and may not be generalizable or representative of practices at non-children's hospitals or those outside the USA. Third, it is possible that increased neuromonitoring reflects the contribution of neurology consultants; the PHIS dataset does not provide information about use of neurology consultation services and whether centers have protocolized neuromonitoring or it is clinician-dependent.

## Conclusions

Clinical teams have used many modalities to screen, diagnose, and monitor for neurologic injury in this high-risk population with minimal evidence to guide their application. This has resulted in significant variation among centers providing ECMO support and between patient populations. There is an opportunity for prospective studies and national organizations to identify patients at the highest risk and provide guidance on best practices for screening and monitoring. Importantly, the identification of injury is essential but insufficient in the comprehensive care of a patient with a brain injury. As we develop best practices for monitoring, we also need to support the multidisciplinary teams, including pediatric intensivists, neurologists, and rehabilitation specialists to develop plans to treat evolving injury and engage children in long-term neurodevelopmental support.

## Supplementary Information

**Additional file 1.** Supplementary digital content.

## Data Availability

The Pediatric Health Information System database is available through a data request to the Children’s Hospital Association (https://www.childrenshospitals.org/content/analytics/product-program/pediatric-health-information-system).

## Abbreviations

CT: Computed tomography
CTC: Clinical transaction classification
ECMO: Extracorporeal membrane oxygenation
EEG: Electroencephalography
MRI: Magnetic resonance imaging
PHIS: Pediatric Health Information System
RACHS-2: Risk adjustment for congenital heart surgery-2
TCD: Transcranial Doppler

## References

[1] Barbaro RP, Brodie D, MacLaren G (2021). Bridging the gap between intensivists and primary care clinicians in extracorporeal membrane oxygenation for respiratory failure in children: a review. JAMA Pediatr.

[2] Dalton HJ, Reeder R, Garcia-Filion P, Holubkov R, Berg RA, Zuppa A (2017). Factors associated with bleeding and thrombosis in children receiving extracorporeal membrane oxygenation. Am J Respir Crit Care Med.

[3] Cook RJ, Rau SM, Lester-Pelham SG, Vesper T, Peterson Y, Adamowski T (2020). Electrographic seizures and brain injury in children requiring extracorporeal membrane oxygenation. Pediatr Neurol.

[4] Yuliati A, Federman M, Rao LM, Chen L, Sim MS, Matsumoto JH (2020). Prevalence of seizures and risk factors for mortality in a continuous cohort of pediatric extracorporeal membrane oxygenation patients. Pediatr Crit Care Med.

[5] Hassumani DO, Shan M, Mastropietro CW, Wing SE, Friedman ML (2022). Seizures in children with cardiac disease on extracorporeal membrane oxygenation. Neurocrit Care.

[6] Bauer Huang SL, Said AS, Smyser CD, Lin JC, Guilliams KP, Guerriero RM (2021). Seizures are associated with brain injury in infants undergoing extracorporeal membrane oxygenation. J Child Neurol.

[7] Bembea MM, Felling R, Anton B, Salorio CF, Johnston MV (2015). Neuromonitoring during extracorporeal membrane oxygenation: a systematic review of the literature. Pediatr Crit Care Med.

[8] Bembea MM (2013). Neuromonitoring of neonatal extracorporeal membrane oxygenation patients using serial cranial ultrasounds. Pediatr Crit Care Med.

[9] Raets MM, Dudink J, Ijsselstijn H, van Heijst AF, Lequin MH, Houmes RJ (2013). Brain injury associated with neonatal extracorporeal membrane oxygenation in the Netherlands: a nationwide evaluation spanning two decades. Pediatr Crit Care Med.

[10] Herman ST, Abend NS, Bleck TP, Chapman KE, Drislane FW, Emerson RG (2015). Consensus statement on continuous EEG in critically ill adults and children, Part I. J Clin Neurophysiol.

[11] Herman ST, Abend NS, Bleck TP, Chapman KE, Drislane FW, Emerson RG (2015). Consensus statement on continuous EEG in critically Ill adults and children, Part II. J Clin Neurophysiol.

[12] Chenouard A, Toulgoat F, Rolland A, Liet JM, Maminirina P, Joram N (2021). Right watershed cerebral infarction following neck cannulation for veno-arterial extracorporeal membrane oxygenation in pediatric septic shock: a case series. Perfusion.

[13] O'Brien NF, Buttram SDW, Maa T, Lovett ME, Reuter-Rice K, LaRovere KL (2019). Cerebrovascular physiology during pediatric extracorporeal membrane oxygenation: a multicenter study using transcranial doppler ultrasonography. Pediatr Crit Care Med.

[14] Salna M, Ikegami H, Willey JZ, Garan AR, Cevasco M, Chan C (2019). Transcranial Doppler is an effective method in assessing cerebral blood flow patterns during peripheral venoarterial extracorporeal membrane oxygenation. J Card Surg.

[15] Pediatric Health Information System (PHIS). Children’s Hospital Association.

[16] Allen P, Zafar F, Mi J, Crook S, Woo J, Jayaram N (2022). Risk stratification for congenital heart surgery for ICD-10 administrative data (RACHS-2). J Am Coll Cardiol.

[17] Hsieh MT, Hsieh CY, Tsai TT, Wang YC, Sung SF (2020). Performance of ICD-10-CM diagnosis codes for identifying acute ischemic stroke in a national health insurance claims database. Clin Epidemiol.

[18] Hsieh MT, Huang KC, Hsieh CY, Tsai TT, Chen LC, Sung SF (2021). Validation of ICD-10-CM diagnosis codes for identification of patients with acute hemorrhagic stroke in a national health insurance claims database. Clin Epidemiol.

[19] Smith JR, Jones FJS, Fureman BE, Buchhalter JR, Herman ST, Ayub N (2020). Accuracy of ICD-10-CM claims-based definitions for epilepsy and seizure type. Epilepsy Res.

[20] Westergren H, Marell Hesla H, Altman M, Wickstrom R (2022). Validation of central nervous system-induced seizures and other neurological variables in the Swedish neonatal quality register. Acta Paediatr.

[21] Feudtner C, Feinstein JA, Zhong W, Hall M, Dai D (2014). Pediatric complex chronic conditions classification system version 2: updated for ICD-10 and complex medical technology dependence and transplantation. BMC Pediatr.

[22] Cuzick J (1985). A wilcoxon-type test for trend. Stat Med.

[23] Wayne MT, Seelye S, Molling D, Hogan CK, Valley TS, Arenberg DA (2022). Variation in U.S. hospital practices for bronchoscopy in the intensive care unit. Ann Am Thorac Soc.

[24] Wayne MT, Seelye S, Molling D, Wang XQ, Donnelly JP, Hogan CK (2021). Temporal trends and hospital variation in time-to-antibiotics among veterans hospitalized with sepsis. JAMA Netw Open.

[25] Wong TE, Nguyen T, Shah SS, Brogan TV, Witmer CM (2016). Antithrombin concentrate use in pediatric extracorporeal membrane oxygenation: a multicenter cohort study. Pediatr Crit Care Med.

[26] Ruth A, McCracken CE, Fortenberry JD, Hall M, Simon HK, Hebbar KB (2014). Pediatric severe sepsis: current trends and outcomes from the pediatric health information systems database. Pediatr Crit Care Med.

[27] Lin J-J, Banwell BL, Berg RA, Dlugos DJ, Ichord RN, Kilbaugh TJ (2017). Electrographic seizures in children and neonates undergoing extracorporeal membrane oxygenation. Pediatr Crit Care Med.

[28] Okochi S, Shakoor A, Barton S, Zenilman AR, Street C, Streltsova S (2018). Prevalence of seizures in pediatric extracorporeal membrane oxygenation patients as measured by continuous electroencephalography. Pediatr Crit Care Med.

[29] Glass HC, Wusthoff CJ, Shellhaas RA (2013). Amplitude-integrated electro-encephalography: the child neurologist's perspective. J Child Neurol.

[30] Abend NS, Topjian AA, Williams S (2015). Could EEG monitoring in critically Ill children Be a cost-effective neuroprotective strategy?. J Clin Neurophysiol.

[31] Abend NS, Topjian AA, Williams S (2015). How much does it cost to identify a critically Ill child experiencing electrographic seizures?. J Clin Neurophysiol.

[32] Ijsselstijn H, Schiller RM, Holder C, Shappley RKH, Wray J, Hoskote A (2021). Extracorporeal life support organization (ELSO) guidelines for follow-up after neonatal and pediatric extracorporeal membrane oxygenation. ASAIO J.

[33] Farhat A, Li X, Huet B, Tweed J, Morriss MC, Raman L (2021). Routine neuroimaging: understanding brain injury in pediatric extracorporeal membrane oxygenation. Crit Care Med.

[34] Guerguerian AM, Vargas-Gutierrez M, Laughlin S (2022). Why clinicians should adopt routine neuroimaging after extracorporeal membrane oxygenation. Crit Care Med.

[35] Ankola AA, Bailly DK, Reeder RW, Cashen K, Dalton HJ, Dolgner SJ (2021). Risk factors associated with bleeding in children with cardiac disease receiving extracorporeal membrane oxygenation: a multi-center data linkage analysis. Front Cardiovasc Med.

[36] Cvetkovic M, Chiarini G, Belliato M, Delnoij T, Zanatta P, Taccone FS (2021). International survey of neuromonitoring and neurodevelopmental outcome in children and adults supported on extracorporeal membrane oxygenation in Europe. Perfusion.

[37] Krishnan S, Schmidt GA (2019). Hemodynamic monitoring in the extracorporeal membrane oxygenation patient. Curr Opin Crit Care.

[38] Lin N, Flibotte J, Licht DJ (2018). Neuromonitoring in the neonatal ECMO patient. Semin Perinatol.

[39] Ong CS, Etchill E, Dong J, Shou BL, Shelley L, Giuliano K (2021). Neuromonitoring detects brain injury in patients receiving extracorporeal membrane oxygenation support. J Thorac Cardiovasc Surg.

