# Supplementary material for: Variation in electroencephalography and neuroimaging for children receiving extracorporeal membrane oxygenation
Source: Crit Care. 2023 Jan 17;27:23. doi: 10.1186/s13054-022-04293-6 (PMC9847194; doi:10.1186/s13054-022-04293-6)
Supplement: Supplementary file 1 — Additional file 1. Supplementary digital content. [file 13054_2022_4293_MOESM1_ESM.docx]

**Variation in Electroencephalography and Neuroimaging for Children Receiving Extracorporeal Membrane Oxygenation**

**Supplemental Digital Content**

Joseph G. Kohne MD MSc^1,2^, Graeme MacLaren MBBS^3^, Renée A Shellhaas MD MS^4^, Giulia Benedetti MD^5^, Ryan Barbaro MD MSc^1,2^

^1^Division of Pediatric Critical Care Medicine, Department of Pediatrics, University of Michigan

^2^Susan B. Meister Child Health Evaluation and Research Center, University of Michigan School of Medicine, Ann Arbor, Michigan

^3^Cardiothoracic Intensive Care Unit, National University Health System, Singapore

^4^Division of Pediatric Neurology, Department of Pediatrics, University of Michigan

^5^Department of Neurology, Seattle Children's Hospital and University of Washington

**Correspondence**: Joseph G. Kohne, M.D. 1500 E. Medical Center Drive, F-6790

Ann Arbor, MI 48109, Email: [jkohne@med.umich.edu](mailto:jkohne@med.umich.edu) Tel: (734) 764-5302 Fax: 734-232-4683

No reprints are planned.

| **Supplemental Table 1: Administrative codes used in the analysis** | | |
| --- | --- | --- |
| Variable | Format | Codes |
| Extracorporeal Membrane Oxygenation | ICD-10PCS | 5A15223; 5A1522F; 5A1522G; 5A15A2F; 5A15A2G; 5A1522H; 5A15A2H |
| Magnetic Resonance Imaging | CTC | 411052 |
| Transcranial Doppler | CTC | 411045, 411046 |
| Computed Tomography | CTC | 411051 |
| Cranial Ultrasound | CTC | 411040, 411041, 411042 |
| Electroencephalography | CTC | 515001 |
| Seizures | ICD-10CM | G40.x, R56.9, P90 |
| Stroke | ICD-10CM | I60.x, I61.x, I62.x I63.x, I64.x, G46.x |
| Antiepileleptic medication |  |  |
| Brivaracetam | CTC | 116002 |
| Cannabidiol | CTC | 116069 |
| Carbamazepine | CTC | 116035 |
| Clobazam | CTC | 116034 |
| Ethosuximide | CTC | 116025 |
| Felbamate | CTC | 116041 |
| Fosphenytoin sodium | CTC | 116015 |
| Lacosamide | CTC | 116023 |
| Lamotrigine | CTC | 116051 |
| Levetiracetam | CTC | 116001 |
| Other specified anticonvulsants | CTC | 116099 |
| Oxcarbazepine | CTC | 116005 |
| Pentobarbital sodium | CTC | 114015 |
| Perampanel | CTC | 116024 |
| Phenobarbital (sodium) | CTC | 114016 |
| Phenytoin (extended) (prompt) (sodium) | CTC | 116021 |
| Rufinamide |  | 116028 |
| Topiramate | CTC | 116061 |
| Valproic acid and derivatives | CTC | 116067 |
| Vigabatrin | CTC | 116063 |
| Zonisamide | CTC | 116007 |
| ICD-10PCS- International Classification of Diseases, Tenth Revision, Procedure Coding System; ICD-10-CM- International Classification of Diseases, Tenth Revision, Clinical Modification;; CTC- Clinical Transaction Classification | | |

| **Supplemental Table 2: Frequency of EEG/Neuroimaging modalities among those who survived to hospital discharge, stratified by age group and etiology** | | | | | | | |
| --- | --- | --- | --- | --- | --- | --- | --- |
|  |  | Age group | | | Etiology | | |
| Modality | Total | Children | Neonates | p | Non-cardiac surgery | Cardiac Surgery | p |
|  | N=5,345 | N=3,109 | N=2,236 |  | N=3,447 | N=1,898 |  |
| Magnetic Resonance Imaging | 2,696 (50%) | 1,195 (38%) | 1,501 (67%) | <0.001 | 1,961 (57%) | 735 (39%) | <0.001 |
| Transcranial Doppler | 363 (7%) | 165 (5%) | 198 (9%) | <0.001 | 230 (7%) | 133 (7%) | 0.64 |
| Computed Tomography | 1,618 (30%) | 1,314 (42%) | 304 (14%) | <0.001 | 880 (26%) | 738 (39%) | <0.001 |
| Cranial Ultrasound | 3,385 (63%) | 1,179 (38%) | 2,206 (99%) | <0.001 | 2,012 (58%) | 1,373 (72%) | <0.001 |
| Under age 1 (n=3431) | 3318 (97%) | -- | -- | -- | 1969 (97%) | 1349 (97%) | 0.84 |
| Electroencephalography | 3,178 (59%) | 1,926 (62%) | 1,252 (56%) | <0.001 | 1,957 (57%) | 1,221 (64%) | <0.001 |

| Supplemental Table 3: Frequency of Neuromonitoring/Neuroimaging modalities among those that did and did not survive to hospital discharge | | | | |
| --- | --- | --- | --- | --- |
|  | Total | Survivors | Non-survivors | p-value |
|  | N=8,746 | N=5,345 | N=3,401 |  |
| Magnetic resonance imaging | 3,130 (36%) | 2,696 (50%) | 434 (13%) | <0.001 |
| Transcranial doppler | 667 (8%) | 363 (7%) | 304 (9%) | <0.001 |
| Computed tomography | 3,267 (37%) | 1,618 (30%) | 1,649 (48%) | <0.001 |
| Cranial ultrasound | 5,510 (63%) | 3,385 (63%) | 2,125 (62%) | 0.42 |
| Electroencephalogram | 5,450 (62%) | 3,178 (59%) | 2,272 (67%) | <0.001 |

| Supplemental Table 4: Frequency of electroencephalography by age group in cardiac surgery and non-cardiac surgery hospitalizations | | | | |
| --- | --- | --- | --- | --- |
|  | Total | Non-cardiac surgery | Cardiac Surgery | p-value |
| Neonate (<29days) | 2061 (58%) | 1283 (54%) | 1108 (68%) | <0.001 |
| 29d to <1 year | 1077 (68%) | 531 (67%) | 546 (70%) | 0.22 |
| 1 year to 4 years | 769 (68%) | 537 (67%) | 232 (71%) | 0.26 |
| 5 years to 12 years | 575 (62%) | 442 (61%) | 133 (64%) | 0.37 |
| 13 years or greater | 638 (58%) | 506 (57%) | 132 (59%) | 0.64 |

| Supplemental Table 5: Multilevel Logistic Regression models for receipt of each modality | | | | |
| --- | --- | --- | --- | --- |
|  |  |  |  |  |
|  | Transcranial Doppler | Computed Tomography | Electro-encephalography | Magnetic Resonance Imaging |
| Discharge Year | 1.05 [0.98,1.13] | 1.05^***^ [1.02,1.08] | 1.25^***^ [1.21,1.29] | 1.05^**^ [1.02,1.08] |
| Age (years) | 0.87^***^ [0.85,0.89] | 1.06^***^ [1.06,1.07] | 0.99^***^ [0.98,0.99] | 0.93^***^ [0.92,0.94] |
| Duration of ECMO support (days) | 1.01^*^ [1.00,1.02] | 1.02^***^ [1.02,1.03] | 1.02^***^ [1.01,1.02] | 1.00 [0.99,1.00] |
| Cardiac Surgery Hospitalization | 1.08 [0.85,1.37] | 1.86^***^ [1.69,2.06] | 1.58^***^ [1.42,1.76] | 0.56^***^ [0.50,0.62] |
| Died during the hospitalization | -- | -- | -- | 0.13^***^ [0.11,0.14] |
| Intraclass Correlation Coefficient | 0.703 | 0.139 | 0.302 | 0.086 |
| Table presents odds ratios and 95% confidence intervals in brackets  ^*^ *p* < 0.05, ^**^ *p* < 0.01, ^***^ *p* < 0.001 | | | | |

| Supplemental Table 6: Types of antiseizure medications (ASM) used in children who received and did not receive an electroencephalogram (EEG) | | | | |
| --- | --- | --- | --- | --- |
|  | Total | No EEG | EEG | p-value |
|  | N=8,746 | N=3,296 | N=5,450 |  |
| Any antiseizure medication | 3,195 (37%) | 672 (20%) | 2,523 (46%) | <0.001 |
| Brivaracetam | 2 (0%) | 1 (0%) | 1 (0%) | 1.00 |
| Cannabidiol | 5 (0%) | 0 (0%) | 5 (0%) | 0.16 |
| Carbamazepine | 3 (0%) | 2 (0%) | 1 (0%) | 0.56 |
| Clobazam | 52 (1%) | 4 (0%) | 48 (1%) | <0.001 |
| Ethosuximide | 3 (0%) | 1 (0%) | 2 (0%) | 1.00 |
| Felbamate | 4 (0%) | 1 (0%) | 3 (0%) | 1.00 |
| Fosphenytoin sodium | 506 (6%) | 29 (1%) | 477 (9%) | <0.001 |
| Lacosamide | 118 (1%) | 5 (0%) | 113 (2%) | <0.001 |
| Lamotrigine | 15 (0%) | 4 (0%) | 11 (0%) | 0.44 |
| Levetiracetam | 1,890 (22%) | 210 (6%) | 1,680 (31%) | <0.001 |
| Other specified anticonvulsants | 12 (0%) | 3 (0%) | 9 (0%) | 0.55 |
| Oxcarbazepine | 53 (1%) | 6 (0%) | 47 (1%) | <0.001 |
| Pentobarbital sodium | 726 (8%) | 212 (6%) | 514 (9%) | <0.001 |
| Perampanel | 2 (0%) | 0 (0%) | 2 (0%) | 0.53 |
| Phenobarbital (sodium) | 1,473 (17%) | 292 (9%) | 1,181 (22%) | <0.001 |
| Phenytoin (extended) (prompt) (sodium) | 117 (1%) | 46 (1%) | 71 (1%) | 0.71 |
| Rufinamide | 8 (0%) | 2 (0%) | 6 (0%) | 0.72 |
| Topiramate | 33 (0%) | 4 (0%) | 29 (1%) | 0.002 |
| Valproic acid and derivatives | 63 (1%) | 7 (0%) | 56 (1%) | <0.001 |
| Vigabatrin | 5 (0%) | 1 (0%) | 4 (0%) | 0.66 |
| Zonisamide | 15 (0%) | 3 (0%) | 12 (0%) | 0.19 |
